# Correction: Brominated and Chlorinated Flame Retardants: The San Antonio Statement

**DOI:** 10.1289/ehp.1003088-erratum

**Published:** 2011-01-01

Competing financial interest declarations were not included in editorials published in the December issue of *Environmental Health Perspectives*; the statements are provided below.

L.S. Birnbuam and A. Bergman, authors of the editorial “Brominated and Chlorinated Flame Retardants: The San Antonio Statement” [Environ Health Perspect 118:A514–A515 (2010)], declare they have no actual or potential competing financial interests.

J. DiGangi, A. Blum, A. Bergman, C. deWit, D. Lucas, D. Mortimer, A. Schecter, M. Scheringer, S Shaw, and T Webster, authors of the “San Antonio Statement on Brominated and Chlorinated Flame Retardants” [Environ Health Perspect 118:A516 (2010)], declare they have no actual or potential competing financial interests.

Beginning in January 2011, *EHP* will publish a declaration of competing financial interests for authors of all editorials.

